# Impact of Bacteriophage-Supplemented Drinking Water on the *E. coli* Population in the Chicken Gut

**DOI:** 10.3390/pathogens9040293

**Published:** 2020-04-16

**Authors:** Sophie Kittler, Ruth Mengden, Imke H. E. Korf, Anna Bierbrodt, Johannes Wittmann, Madeleine Plötz, Arne Jung, Tatiana Lehnherr, Christine Rohde, Hansjörg Lehnherr, Günter Klein, Corinna Kehrenberg

**Affiliations:** 1 Institute for Food Quality and Food Safety, University of Veterinary Medicine Hannover, Foundation, Bischofsholer Damm 15, 30173 Hannover, Germany; madeleine.ploetz@tiho-hannover.de; 2 Food Inspection, Animal Welfare and Veterinary Service of the Land of Bremen, Border Control Post Bremerhaven, Senator-Borttscheller-Straße 8, 27568 Bremerhaven, Germany; ruth.mengden@lmtvet.bremen.de; 3 Leibniz Institute DSMZ—German Collection of Microorganisms and Cell Cultures, Inhoffenstraße 7B, 38124 Braunschweig, Germany; ims16@dsmz.de (I.H.E.K.); johannes.wittmann@dsmz.de (J.W.); chr@dsmz.de (C.R.); 4 Institute for Hazardous Materials Research, Waldring 97, 44789 Bochum, Germany; anna.bierbrodt@gmail.com; 5 Clinic for Poultry, University of Veterinary Medicine Hannover, Foundation, Bünteweg 17, 30559 Hannover, Germany; arne.jung@tiho-hannover.de; 6 PTC Phage Technology Center GmbH, Siemensstraße 42, 59199 Bönen, Germany; t.lehnherr@ptc-phage.com (T.L.); h.lehnherr@ptc-phage.com (H.L.); 7 Institute for Veterinary Food Science, Justus-Liebig-University Giessen, Frankfurter Straße 92, 35392 Giessen, Germany; corinna.kehrenberg@vetmed.uni-giessen.de

**Keywords:** *E. coli*, ExPEC, phages, bacteriophages, microbiota, chicken microflora, colibacillosis, multidrug resistance

## Abstract

Among intestinal coliform microbes in the broiler gut, there are potentially pathogenic *Escherichia* (*E.*) *coli* that can cause avian colibacillosis. The treatment with antibiotics favors the selection of multidrug-resistant bacteria and an alternative to this treatment is urgently required. A chicken model of intestinal colonization with an apathogenic model strain of *E*. *coli* was used to test if oral phage application can prevent or reduce the gut colonization of extraintestinal pathogenic *E. coli* variants in two individual experiments. The *E. coli* strain E28 was used as a model strain, which could be differentiated from other *E. coli* strains colonizing the broiler gut, and was susceptible to all cocktail phages applied. In the first trial, a mixture of six phages was continuously applied via drinking water. No reduction of the model *E. coli* strain E28 occurred, but phage replication could be demonstrated. In the second trial, the applied mixture was limited to the four phages, which showed highest efficacy in vitro. *E. coli* colonization was reduced in this trial, but again, no reduction of the *E. coli* strain E28 was observed. The results of the trials presented here can improve the understanding of the effect of phages on single strains in the multi-strain microbiota of the chicken gut.

## 1. Introduction

If management practices fail to prevent disease in commercially reared poultry, birds are routinely treated with antibiotics such as fluoroquinolones, colistin, or sulfamethoxazole/trimethoprim [1]. As they have an antimicrobial effect on all susceptible microbes of the natural gut microbiota, they pose increased selective pressure, favoring the growth and spread of resistant bacteria [2]. Antimicrobial resistances are a serious concern for human health, and all of the above-mentioned antibiotics were ranked as highly or critically important antimicrobials by the World Health Organization in 2017 [3,4]. Resistant *E. coli* variants that occur in poultry production might spread to humans via the food chain or disseminate their resistance determinants [5,6]. Therefore, prudent use of antimicrobial agents in animals should be executed [4].

*E. coli* is an opportunistic pathogen that can cause severe disease in humans and animals, including chickens [6]. Extraintestinal pathogens such as avian pathogenic *E. coli* (APEC) colonize the chicken gut without causing symptoms. Inhalation of dust from feces is suggested to be the most frequent way of infection [7,8]. Prevention of intestinal colonization might, therefore, prevent infection and clinical signs of the disease. Clinical signs include septicemia and fibrinous lesions of the organs (airsacculitis, pericarditis, perihepatitis) [6,9]. Approximately 10–15% of intestinal coliform microbes in the broiler gut are potentially pathogenic types that can cause symptoms of avian colibacillosis [7]. The occurrence of a limited number of widespread pathogenic *E. coli* clones [10] implies the possibility to use a specific phage cocktail for control purposes against these pathogenic *E. coli* [11,12,13,14].

The use of bacteriophages (phages) is a promising alternative to conventional antibiotic use [15]. Phages were widely used for human therapy of bacterial infections in Eastern Europe and target only bacterial cells [16]. Studies have shown that phages can combat bacteria in the gut environment under commercial rearing conditions [17] and that different ways of application can be used to prevent clinical signs of experimentally induced avian colibacillosis [18,19,20,21,22,23]. Barrow et al. inoculated three-week-old and newly hatched chickens intramuscularly and intracranially with *E. coli* and observed almost 100% mortality without phage therapy [18]. In contrast, birds were protected from disease, if a high dose of a single phage preparation, containing a phage named ‘bacteriophage R’, was administered simultaneously with the pathogenic bacteria [18]. Studies carried out by Huff et al. showed that intramuscular injection of two different phages, SpR02 and DAF6, resulted in a 15% mortality rate, while untreated birds showed a mortality rate of 68% [1]. Other trials that used a three phage cocktail of two myoviruses (phi F78E and phi F61E) and one siphovirus (phi F258E) in a combined oral and spray application in naturally infected flocks decreased the flock mortality to levels below 0.5% in ten out of eleven investigated flocks [24]. However, these phage application setups cannot be applied easily in commercial broiler production, but application of vaccines and antibiotics via drinking water is routinely used in commercial broiler production and can be easily adopted for the application of phages [17]. The applied phages could then target potentially pathogenic serotypes in the chicken gut to prevent inhalation of and subsequent infection by contaminated fecal particles. They could, therefore, forestall the first step of infection by pathogens causing, for example, colibacillosis [17,24].

The phages EW2, AB27, G28, TB49, KRA2, and TriM that were used in this study all belong to the virus family *Myoviridae* and represent members of the morphotypes A1 (EW2, AB27, KRA2), A2 (G28, TB49), and A3 (TriM) [25]. TB49 lysed 50% of the 66 host strains tested, while the host range of the other phages ranged from 12% to 29%. Reproduction and release of progeny took 20 to 40 min in vitro, depending on the individual phage. The applied cocktail was selected based on its high lytic efficacy in broth culture. Including only the four phages that proved most effective in vitro and deleting the phages KRA2 and TriM resulted in the four-phage cocktail that was used in the second trial [25].

Other studies successfully used cocktails in murine models for intestinal reduction of *E*. *coli* composed of three phages representing different families of the order *Caudovirales*: *Siphoviridae*, *Myoviridae*, and *Podoviridae* [26,27]. Cocktails that are composed of phages with different receptors or infection mechanisms were suggested to reduce the occurrence of resistant bacteria during phage application and to ensure effectiveness of the preparation under a broad variety of conditions [28,29].

In this study, our aim was to test whether the oral application of phage cocktails can prevent gut colonization of the model *E. coli* strain E28 in chicken flocks. It was intended to use a model strain that was trackable by more than one phenotypic marker in order to consider potential reverse mutation of a single marker. In the case of our model strain E28, these properties are its resistance to kanamycin and the ability to grow in the presence of potassium tellurite. The present study includes two animal experiments: (i) in the first trial, we investigated a six-phage cocktail applied at a low multiplicity of infection (MOI) (in the following sections, referred to as the six-phage trail); and (ii) in the second trial, we investigated a four-phage cocktail lacking phages KRA2 and TriM, but applied at a high MOI (in the following sections, referred to as the four-phage trail).

The results of these first trials on the reduction of *E*. *coli* in a chicken model can improve the understanding of the effect of phages on single strains in the multi-strain microbiota of the chicken gut.

## 2. Results

Both trials were conducted to examine the prophylactic effect of phage application on the colonization of the model *E. coli* strain E28 in broiler chickens. Phages were applied via drinking water from day one post hatch until the end of the experiment. The model *E. coli* strain E28 was orally inoculated on day six or seven post hatch, respectively. Inoculation doses of the model *E. coli* strain E28 differed between the trials and a higher concentration of phages was supplemented in the four-phage trial to achieve a high MOI. The cocktail used in the four-phage trial was adjusted by deleting the phages KRA2 and TriM. To examine the effect of the cocktail on the overall *E. coli* load in the chicken gut and detect possible interactions of the *E*. *coli* gut microbiota and the target strain, concentrations of total fecal *E. coli* were determined in addition to the model strain in all fecal samples.

### 2.1. No Reduction of E. coli in Feces Occurred during the Six-Phage Trial

**Figure 1:**
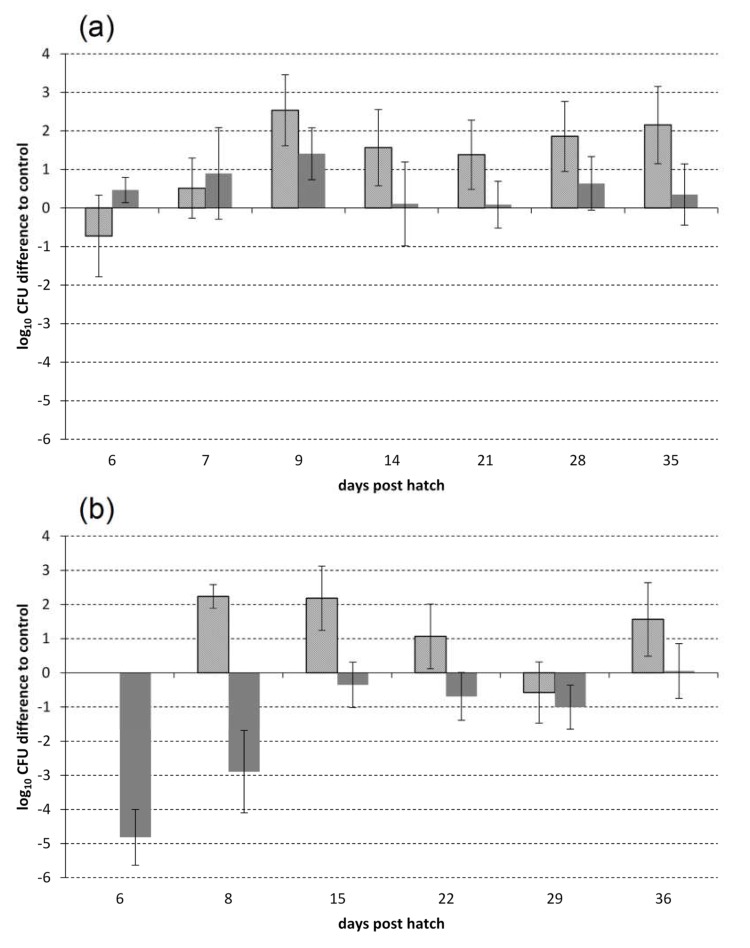
Mean difference of bacterial concentrations in the experimental group compared with the control. Drinking water of the experimental group was supplemented with a phage cocktail in both experiments, containing six (**a**) or four (**b**) phages, respectively. Counts in the control group resemble the baseline. Patterned bars represent mean differences in fecal concentrations of the model strain *E. coli* E28, while grey bars represent mean differences of fecal *E. coli*. Error bars show confidence intervals of the difference between the means of the control and experimental groups. Differences are significant if confidence intervals include the baseline. (**a**) Six-phage trial (cocktail containing the phages TriM, AB27, EW2, TB49, KRA2, and G28), (**b**) four-phage trial (cocktail containing the phages AB27, EW2, TB49, and G28). CFU Colony forming units.

In the six-phage trial, the model strain *E*. *coli* E28 was applied at a concentration of log_10_ 8.99 colony forming units (CFU)/animal. When the first fecal samples were taken three hours later, fecal *E. coli* E28 counts in the experimental group were 0.7 log units lower than in the control group (Figure 1a, patterned bars). However, this difference was not significant and no reduction in the experimental group was observed thereafter. Interestingly, mean concentrations of the model strain in fecal samples of the experimental group were significantly higher than in the control group on day nine post hatch and on all following sampling days (Figure 1a, patterned bars; 9 days post hatch (dph): *p* < 0.0001; 14 dph: *p* = 0.005; 21 dph: *p* = 0.0159; 28 dph: *p* = 0.0004; 35 dph: *p* = 0.0004). The highest mean concentration of the model strain E28 was observed on the day of inoculation. Subsequently, model strain concentrations in the control dropped to relatively stable levels of approximately log_10_ 4 CFU/g feces, while they remained approximately 1.5 log units higher in the experimental group (Table 1).

The results from analyzing all fecal *E. coli* growing on tryptone bile X glucuronide (TBX) agar suggest that no reduction of overall fecal *E*. *coli* occurred in the experimental group during the six-phage trial (Figure 1a, grey bars). No reduction, but a 0.8 log units higher mean concentration was detected in the experimental group compared with the control group on day 6 post hatch (Figure 1a, grey bars; *p* = 0.006). On day 9 post hatch, the mean fecal *E. coli* concentration in the experimental group was still significantly higher (Figure 1a, grey bars; *p* = 0.0007). Mean fecal *E. coli* concentrations from samplings at days 14, 21, 28, and 35 post hatch showed no significant reduction and ranged between 6.3 and 6.9 log_10_ CFU/g in both groups (Table 1).

### 2.2. A Delay in Fecal E. coli Colonization and Lower Fecal E. coli Load Occurred in the Phage Supplemented Group of the Four-Phage Trial

As presented in Figure 1b (grey bars), mean fecal *E. coli* counts were lower in the experimental group compared with the control group during the four-phage trial from 6 dph to 29 dph. In the experimental group, fecal *E. coli* counts did not exceed log_10_ 6 CFU/g feces during the experimental period of 36 days (Table 1). In the control group, fecal *E. coli* counts ranged between log_10_ 6 CFU/g feces and log_10_ 7 CFU/g feces from 6 dph to 36 dph (Table 1). While *E*. *coli* colonized the control group at high levels after six days (Table 1), the experimental group showed a significantly lower fecal *E. coli* concentration on day 6 (*p* < 0.0001) and 8 post hatch (*p* = 0.0012; Figure 1b, grey bars). On day 15 post hatch, mean fecal *E. coli* counts in the experimental group also reached log_10_ 5.9 CFU/g feces, but, in contrast to the control, counts did not rise above log_10_ 6 CFU/g feces during the whole experiment (Figure 1b). A significant difference between the groups occurred on day 29 post hatch (Figure 1b, grey bars, *p* = 0.0072).

The *E. coli* model strain E28 was orally inoculated on day 7 post hatch in the four-phage trial, using a dose of log_10_ 4.67 CFU/animal. Interestingly, colonization of *E. coli* E28 after inoculation established more quickly in the experimental group than in the control (Table 1), and significantly higher levels of colonization were observed (8 dph: *p* = 0.0015; 15 dph: *p* = 0.0003; 22 dph: *p* = 0.04; 36 dph: *p* = 0.0146; Figure 1b, patterned bars). This is in contrast to the results observed for fecal *E. coli*. However, differences between mean counts of *E. coli* E28 were lower than differences in the overall counts of fecal *E. coli* on day 9 and 15, as shown in Figure 1b.

### 2.3. Phage Concentrations in Feces Exceeded the Phage Intake Dose on Individual Days

The detected phage concentrations in feces exceeded the phage intake dose on days 6 and 7 post hatch in the six-phage trial. Mean phage concentrations in fecal samples of this trial varied between log_10_ 4.1 plaque forming units (PFU)/g and log_10_ 7.3 PFU/g, as shown in Table 2. In the four-phage trial, phage concentrations in feces exceeded the phage intake dose on day 15 post hatch. Mean phage concentration from fecal samples of this trial varied between log_10_ 3.5 PFU/g and log_10_ 6.9 PFU/g (Table 2, mean phage counts/intake dose).

### 2.4. No Phage Resistant Subpopulation of the E. coli Model Strain E28 Was Detected

Overall, 203 isolates from TBX agar plates obtained during the six-phage trial and 115 isolates from the four-phage trial were examined for phage susceptibility. Two percent of *E. coli* E28 re-isolates from the phage supplemented group of the six-phage trial showed reduced susceptibility to the cocktail, as presented in Table 3. All isolates from the experimental group of this trial remained susceptible to the phages TB49 and G28 (Table 3). No plaque formation was observed in nine percent of re-isolates on EW2, four percent on KRA2, and three percent on AB 27 (data not shown). Rates of 100-fold reduced plaque formation are shown in Table 3. In the control group of the six-phage trial, all tested re-isolates were fully susceptible to the phages, except two isolates showing a 100-fold reduced plaque formation on TB49 (Table 3).

In the four-phage trial, all re-isolated *E. coli* E28 remained fully susceptible to the phage cocktail (Table 3). Twenty percent of the re-isolates from the experimental group showed reduced susceptibility to EW2, including fifteen percent that showed no plaque formation at all. Ten percent showed reduced susceptibility to TB49, including three percent showing no plaque formation. Seven percent of the re-isolates were fully resistant against AB27, including six percent showing no plaque formation (Table 3). In the control group of the four-phage trial, four percent of the re-isolated *E*. *coli* E28 showed reduced susceptibility to TB49, but all re-isolates were fully susceptible to the other phages (Table 3).

### 2.5. Data Suggest That E. coli E28 Did Not Show Changed Growth Characteristics or Metabolic Changes after Contact with Phages

To investigate whether growth characteristics and metabolite utilization of *E. coli* have changed and influenced population dynamics during the trial, selected isolates from the trials were examined for utilization of carbon sources and growth in liquid medium. Model strain E28 re-isolates were selected based on the results of phage susceptibility testing. The respective patterns of susceptibility are shown in Table 4. Additionally, non-E28 *E*. *coli* deriving from the trials were examined. Most of the *E. coli* E28 re-isolates showed few changes compared with the original E28 (Table 4, re-isolates from the six-phage and four-phage trial). The results indicated that the reduction of phage susceptibility was not associated with a specific metabolic profile or changed metabolite utilization. Two re-isolates that exhibited prominent changes in metabolic features showed reduced growth, but carbon source utilization was increased in one and reduced in the other (Table 4, re-isolates 116 and 156 from the six-phage trial).

Non-E28 *E. coli* from the trials showed almost full resistance against the applied phages (Table 4, non-E28 six-phage and four-phage trial). Most of them showed increased growth rates and a certain pattern of increased utilization of carbon sources compared with the model strain (Table 4).

## 3. Discussion

Colibacillosis is a major endemic disease with economic significance for the poultry industry [7]. In broiler flocks, it is a severe disease that can cause high mortality rates and requires treatment. Despite intensive research, vaccination and improved management practices cannot completely prevent infections [13,30]. APEC colonize the chicken gut without causing symptoms and inhalation of dust from feces is suggested to be the most frequent way of infection [7,31]. The prophylactic effect of the phages on intestinal colonization might, therefore, prevent the disease. This could reduce the necessity of using antibiotics that are critically important for human health [4]. We used a model of asymptomatic gut carriage in chickens for the evaluation of a preventive phage cocktail. To our knowledge, this is the first in vivo approach to model reduction of an *E. coli* target strain in the chicken gut. All previous studies on phages against colibacillosis utilized a curative approach. Despite good results in many studies, the application techniques used do not seem feasible in commercial broiler production [1,18,19,20,21,23,24,32]. Additionally, a curative approach does not take into account the importance of preventing economical losses for the farmers before clinical symptoms occur.

Our results indicate that neither the four- nor the six-phage cocktail could induce targeted reduction of the model *E. coli* strain E28. Instead, lower colonization and retarded establishment of total fecal *E. coli* occurred in the phage supplemented group of the four-phage trial.

One reason for the finding that targeted reduction of the model strain E28 did not occur could be that phages were not able to infect and lyse this strain in vivo. However, the results show that phages were detected at levels that indicate phage replication on 6 dph and 7 dph of the six-phage trial and on 15 dph of the four-phage trial (Table 2). The calculated maximum daily intake of phages did not take into account inactivation in the gut and the high proportion of spilled water when using bell drinkers, as these variables are hard to estimate [33]. However, these factors might lead to exaggerated expectations concerning phage concentrations in feces. Thus, mean phage counts equal to the calculated expectations might already indicate phage replication (Table 2, six-phage trial: 9–28 dph, four-phage trial: 22 dph and 29 dph). On the basis of these considerations, we assume that phage infection and replication occurred from the beginning of the six-phage trial and by 15 dph in the four-phage trial at the latest.

In the four-phage trial, overall lower colonization and retarded establishment of fecal *E. coli* occurred in the phage supplemented group compared with the control. Interestingly, colonization of the fully susceptible model strain *E. coli* E28 established faster in the experimental group than in the control. Phage replication mainly relies on the density of phages and availability of the host [34]. In the four-phage trial, the drinking water was supplemented with an overall 2.1 log units higher concentration of the phage cocktail compared with the six-phage trial. Additionally, birds were inoculated with an approximately 4 log units lower concentration of the model *E*. *coli* strain E28. Thus, a higher phage dose per bacterial cell was calculated from the applied concentrations [35], representing a higher MOI in the four-phage trial. However, as stated by Abedon [36], the key value is the MOI_adsorption_, describing the number of phages actually adsorbing per bacterium. This value is dependent on the mass and concentration of the adsorbing particles at the site of infection and might have been higher in the six-phage trial as a consequence of higher bacterial density [36,37]. The model strain was not reduced in our experiments as an increased phage concentration does not necessarily lead to a reduction of bacteria. For efficient reduction of the bacterial load, inactivation of bacteria by the phages has to exceed bacterial growth. We hypothesized that the higher MOI might have had an inhibitory effect on the overall fecal *E. coli*. Phages might have preferred another host rather than the model strain E28. They could have used this strain to replicate while the targeted model strain was hiding in the crypts. This reduction of competing bacteria in the gut might have improved growth of the model strain *E. coli* E28 in the phage-supplemented group [38,39]. Evasion of phage infection by hiding of intestinal *E. coli* strains was observed in previous studies [26]. However, the non-E28 *E. coli* isolates tested in our study showed low susceptibility to the applied phages in vitro (Table 4) and, as *E. coli* E28 was detected in feces, at least some bacterial cells were available for phage infection in the lumen of the gut.

In vitro results show that the six-phage cocktail covered 67% and the four-phage cocktail covered 62% of the 66 *E*. *coli* host strains tested. Another cocktail that was successfully used in mice by Maura et al. showed a narrow host spectrum, lysing only 24% of 59 tested *E*. *coli* strains in vitro [40]. The broad host range of our cocktail could have facilitated phage replication in other intestinal *E*. *coli* strains compared with the model strain. Additionally, the phages most effective for reduction of the model strain might have been diluted by the others if too many phages were included in the cocktail. Some studies used only three phages, but included members of different virus families. They showed a significant reduction of targeted *E*. *coli* strains in murine models of extraintestinal pathogenic *E*. *coli* (ExPEC) infections [26,27,41]. However, the effects were transient in the study of Maura et al., despite an according cocktail composition. Other results comparable to our study did not find any decrease in the bacterial concentrations of targeted *E. coli* after phage application [32,33,42]. One study using a four-phage cocktail of three podoviruses and one myovirus observed no decrease in mortality of chickens showing severe colibacillosis. However, the four phages showed a lytic spectra that was even broader than the host spectrum of our phages (19–61%) [32].

Another explanation for the reduction of other *E. coli* instead of the model strain could be a difference between in vivo and in vitro phage susceptibility, as reported by other authors [43]. A few non-E28 *E*. *coli* isolates from the trials were tested, as shown in Table 4. They showed almost no susceptibility to the phages. However, they might not represent the diversity of *E*. *coli* in the chicken gut, and a study published by De Sordi et al. reported drifts in the genetic diversity and infectivity of bacteriophages in vivo that have not been examined in our study [44]. Additionally, synergies between the host immune system and phages were reported to promote bacterial clearance, especially of virulent bacteria. The immune system might thus have had an additional effect on the colonization of *E*. *coli* strains in our study. In virulent bacteria, reductions can be considered as a trade-off between phage resistances and virulence in bacteria that could be therapeutically useful, but is not taken into account in experimental ExPEC models, using non-pathogenic model strains [45,46].

As shown in Table 2, resistances against the applied phages were moderate or low and only two percent of *E. coli* E28 re-isolates from the six-phage trial showed reduced susceptibility to the cocktail as a whole, confirming the appropriate selection of combined phages for preventing bacterial resistance [29].

We assumed a changed growth characteristic or metabolic alterations of *E. coli* in the phage supplemented group to be responsible for the increased levels of colonization. Changes in the growth and metabolism of the fecal *E. coli* microbiota could create an advantage for the targeted model strain growth owing to competitive interplay. However, growth curves and metabolic characteristics as compared by OmniLog^TM^ phenotypic microarray did not reveal any changes supporting this suggestion. Two *E. coli* E28 re-isolates of the six-phage trial showed reduced growth and different alterations in their metabolic patterns. However, the changes were observed only in two out of eight isolates, while the properties of the remaining six isolates were comparable to the isolate tested from the control (Table 4). The changes observed may be isolate-specific and the results indicated no association between growth in vitro, metabolic changes, and phage susceptibility (Table 4). Thus, an effect of the observed findings on the outcome of the trial is rather unlikely. Colonization of *E. coli* and the gut microflora relies on many different factors that cannot completely be monitored [38,39]. An impact of the gut flora on the growth of individual strains is of course possible and is the most probable explanation for our results. Despite similar rearing conditions, microbial communities in poultry were found to be different even when litter was reused [47]. An increasing understanding of the interplay between individual strains and commensal microbiota will help to better understand the effect of phages in this environment.

Maura et al. treated enteroaggregative *E. coli* (EAEC) in a mouse model with full lytic in vitro activity of the phages. The applied cocktail was composed of the three phages CLB_P1 (*Podovirus*), CLB_P2 (*Myovirus*), and CLB_P3 (*Siphovirus*) [26]. The authors found growth rates and susceptibility in fecal samples to be different compared with ileal samples. A weaker decrease of the targeted *E. coli* 0104:H4 55989Str model strain occurred in fecal samples compared with ileal samples of mice [26]. These findings imply that measurement of *E. coli* E28 load from other intestinal compartments might have resulted in other outcomes of our trial. However, as bacteria from feces attached to dust particles are crucial for infection of the respiratory tract, reductions in other compartments of the gut do not have any significance for our issue.

In most of the murine models, mice were fed with antibiotics to disrupt the normal intestinal flora [27,41]. This is a difference to our trial, where the normal intestinal flora was not disrupted by antibiotics. It is, however, likely that this flora was modulated by the phages in the drinking water in our study. The interaction between phages and bacteria is much more complex in the gut compared with in other organs [48,49]. This might be especially true when a high diversity of commensal bacterial strains colonizes the gut. Depending on different environments in the trials, the localization or expression of the bacteriophage receptor on the cell surface might have differed [48]. As stated by some authors, pseudolysogeny may occur under specific environmental conditions and affect the viral cycle of the applied phages [48]. Maura et al. suggested a pseudolysogenic state of their phages in the large intestine having consequences for colonization [26]. In contrast to chicken, *E. coli* strains are not generally considered to be part of the intestinal flora in mice [50], which has been the model organism in all other trials with more promising results using a targeted model-strain-approach [48]. One could thus assume that the chicken gut, where multiple *E. coli* strains are natural commensals, is a more complicated environment for targeted *E. coli* reduction than the mouse intestine, where *E. coli* is not commonly observed.

To summarize, in this study, we contribute to the knowledge on targeted phage application in chickens. To our knowledge, this is the first trial on the effect of targeted phage application in an *E. coli* multi-strain environment of chickens. As previous authors have pointed out, generalizations from a single model are hardly possible [26,32]. Our results show that targeted reduction of intestinal *E. coli* strains in a multi-strain environment of commensals is difficult to achieve. However, our findings on phage resistances, metabolic activity, and growth of re-isolates from fecal samples suggest that the interplay of bacterial strains in the microbiota of chickens plays an important role in this context and contributes to a better understanding of the effect of phages on single bacterial strains in a multi-strain environment.

## 4. Materials and Methods

### 4.1. Animal Trials

#### 4.1.1. Ethical Statement

The animal study (title: Application of Phages for the Prevention of Colibacillosis—Proof of Principle) was approved by the local ethic committee of the federal state of Lower Saxony (LAVES, Niedersächsisches Landesamt für Verbraucherschutz und Lebensmittelsicherheit), accession number 33.12-42502-04-16/2271.

#### 4.1.2. Study Design and Experimental Procedures

Six-phage trial: One control and one experimental group were included in the experiment. Per group, sixteen freshly hatched Ross 308 broilers were housed until 35 days post hatch (dph). Drinking water in the experimental group was supplemented with phages during the whole experiment; the supplemented water was changed daily. Phage concentrations in water samples were analyzed regularly. Fresh fecal samples were taken from all birds of the control and the experimental group on the day of placement (0 dph) and on days 6, 7, 9, 14, 21, 28, and 35 post hatch by placing the birds in a clean bucket until defecation. Samples were examined for bacteria and phages as described below. Enumeration of the model strain *E. coli* E28 and fecal *E. coli* started after oral inoculation with the model strain on day 6 post hatch. Phage concentrations were measured on 6, 7, 9, 21, and 35 dph. On day 35 post hatch, birds were sacrificed.

Four-phage trial: One control and one experimental group were included in the experiment. Sixteen Ross 308 broilers per group were housed in accordance with the six-phage trial, but until 36 dph. Consistent with the six-phage trial, drinking water was supplemented with phages during the whole experiment. The supplemented water was changed daily and examined regularly, as stated above. Fresh fecal samples were taken from all birds of the control and the experimental group on the day of housing (0 dph) and on days 6, 8, 15, 22, 29, and 36 post hatch, as described for the six-phage trial. Samples were examined for *E. coli* counts and phages as described below. Enumeration of the model strain *E. coli* E28 started 8 dph, 24 h after oral inoculation at day 7 post hatch. Enumeration of fecal *E. coli* started 6 dph and phage concentrations in fecal samples were measured on 0, 8, 15, 22, 29, and 36 dph. On day 36 post hatch, birds were sacrificed.

#### 4.1.3. Housing and Care

For both animal experiments, chicks from a commercial hatchery were transferred to the animal housing facilities of the Clinic for Poultry of the University of Veterinary Medicine Hannover, Foundation. The birds were divided into two groups (experimental and control group). Each group was placed in one separate unit of the isolation facility in both experiments.

Commercial complete feed and municipal water in bell drinkers were supplied ad libitum. Water in the experimental group was supplemented with the respective phage cocktail as described below. Wood shavings were used as litter (approximately 22 kg/m^2^) and the broilers were kept at a stock density below three animals/m^2^. Drinking water consumption was measured by weighing drinking water input and residues on each day of the trial. Drinking water consumption for each bird was calculated by dividing the total consumption volume by the number of birds. Two animal keepers took care of the two separate groups.

### 4.2. Laboratory Procedures

#### 4.2.1. Bacteriophage Cocktail Composition and Concentration in Drinking Water

A well typed phage cocktail was composed for each experiment, as described by Korf et al. [25]. Briefly, phages were isolated from manure and surface water, using *E*. *coli* isolates from animal origin, laboratory strains, and isolates from the ECOR collection. Subsequently, the phages were thoroughly characterized on a broad collection of 66 *E. coli* strains. TB49 lysed 50% of the tested *E*. *coli* strains, while the host range of the other phages ranged from 12% to 29%. Reproduction and release of progeny took 20 to 40 min in vitro, depending on the individual phage.

The applied cocktail was selected based on its high lytic efficacy in broth culture and results from in vitro experiments on the occurrence of phage-resistant mutants. Even though some cross resistances occurred, none of the isolates were resistant against all phages. Subsequently, the included phages were further characterized. The phages all belong to the virus family *Myoviridae* and represent members of the morphotypes A1 (EW2, AB27, KRA2), A2 (G28, TB49) and A3 (TriM) [25].

The G28 genome sequence was deposited at NCBI GenBank under the accession number MG867727.

The phage cocktail for the six-phage trial was composed of the six phages AB27, TB49, G28, TriM, KRA2, and EW2 in equal parts. The average concentration of phages in the drinking water samples was log_10_ 4.6 plaque forming units (PFU)/mL.

Including only the four phages that proved most effective in vitro and deleting the phages KRA2 and TriM resulted in the four-phage cocktail that was used in the second trial [25]. Accordingly, the cocktail for the four-phage trial was composed of the phages AB27, TB49, G28, and EW2 in equal parts. The drinking water samples contained a mean phage concentration of log_10_ 6.7 PFU/mL.

#### 4.2.2. Selection of *E. coli* Model Strain E28 and Inoculation of Animals

Out of a collection of *E. coli* strains isolated from poultry meat, the commensal *E. coli* E28 field strain was chosen as model strain for examining targeted reduction by phages in animal experiments [25]. Asymptomatic colonization of the gut and quantitative tracking were essential properties for its selection. *E. coli* E28 expressed resistance to kanamycin and, to confirm the genomic location of the resistance gene *aphA,* its genome was completely sequenced (PMID: 28572305) [51]. The resistance marker and the additional capability to grow in the presence of potassium tellurite allowed the preparation of a selective agar for the re-isolation of *E. coli* E28 from feces.

Birds were orally inoculated with the model strain *E. coli* E28 to initiate intestinal colonization. The inoculation dose was log_10_ 8.9 colony forming units (CFU)/animal in the six-phage trial and log_10_ 4.67 CFU/animal in the second trail.

#### 4.2.3. Bacterial Enumeration

To determine the colony forming units (CFU) of *E. coli* model strain E28 and fecal *E. coli*, the pour plate method was used. Fecal samples from the experiments were immediately processed after sampling. Procedures regarding the pouring of plates, incubation time, and counting of colonies were performed according to the recommendations of ISO 16649-2 for the enumeration of beta-glucuronidase-positive *E. coli*. For the first step of a ten-fold dilution series, approximately 1 g of feces was 1:10 diluted in SM-buffer (50 mM Tris-HCl, 8 mM magnesium sulfate, 100 mM sodium chloride, and 0.01% gelatin, pH 7.5). Subsequent dilutions were prepared using sodium chloride peptone buffer (5.8 g/L NaCl, 1 g/L peptone). One milliliter of each dilution was poured into petri dishes in duplicate. Tryptone bile X glucuronide (TBX) agar (Carl Roth GmbH & co KG, Germany) was used for the cultivation of fecal *E. coli*. For the selective growth of *E. coli* E28, the agar was supplemented with kanamycin (0.1 g/L, Carl Roth GmbH & co KG, Germany) and potassium tellurite (2.5 mg/L, Sigma-Aldrich Inc., US) [52,53]. Plates were incubated under aerobic conditions for 20 ± 2 h at 44 °C for *E. coli* and 42 °C for *E. coli* E28.

To confirm *E. coli* E28 on selective agar plates, one colony of each sample was tested by PCR-based serotyping. Colonies were heated in distilled water at 95 °C for 15 minutes and centrifuged at 13,000 rpm for five minutes. Total genomic DNA was obtained from the supernatant. A specific PCR assay targeting the fliC H34 gene (accession number AY2500161) was used to identify *E. coli* E28 isolates. Primers forward (5′-acc ggg act aac tct gat tcg-3′) and reverse (5′-cac caa tag taa ctg atg cag c-3′) were used to amplify an internal segment of the gene (94 °C, 3 min; 94 °C, 0.5 min; 52 °C, 0.5 min; 72 °C, 45 s; 35 cycles; 72 °C, 5 min; 4 °C, pause). Subsequently, gel electrophoresis was used to visualize amplified DNA. In order to exclude false negative results of very small colonies, all isolates with negative results were again cultivated and serotyped by PCR after DNA isolation using the DNeasy Blood & Tissue Kit (QIAGEN, Hilden, Germany) according to the manufacturer‘s instructions.

#### 4.2.4. Bacteriophage Enumeration

The soft-agar overlay plating method was used to determine the number of phages in the samples, as described previously [54], with the modification of using LB broth as nutritional medium (Carl Roth GmbH & co KG, Germany). The tenfold dilution of the samples in SM-buffer, which was used for bacterial enumeration before, was shaken overnight at 4 °C. The samples were centrifuged at 4700× *g* and the supernatant was filtrated through a 0.2 µm polyether-sulfone membrane (Carl Roth GmbH & co KG, Germany). A serial dilution of the filtrate in SM-buffer was prepared and plated in duplicate, using an overnight culture of *E. coli* E28. Plates were incubated at 37 °C for 24 h.

#### 4.2.5. Phage Susceptibility Testing

To determine the phage susceptibility of re-isolates, colonies were picked from TBX agar and supplemented TBX agar plates of each sample. Re-isolates were stored in 10% skim milk at 20 °C until further examination.

In the six-phage trial, microplate analysis was used as a screening method, combined with efficiency of plating (EOP) analysis for re-isolates, which did not show plaque formation in the microplate test. Overall, 109 re-isolates from the control and 94 re-isolates from the experimental group of the six-phage trial were tested using microplate analysis, resulting in 1421 single tests. Analysis was performed as described by Fischer et al. [55], with modifications regarding the nutrient medium, bacterial inoculum density, and concentration of phage test dilutions. A McFarland standard of 0.5 was prepared from blood agar overnight cultures of the bacterial re-isolates and 10 µL of these suspensions was plated together with 10 µL of the phage solution in 0.5 mL of LB overlay agar (0.7% agar agar). The concentration of the phage solutions ranged between log_10_ 4 and 6 PFU/mL, depending on the plaque size of the corresponding phage. For testing of the phages AB27 and TB49, the overlay method was used to improve the visibility of very small plaques. The overlays were prepared as described previously [55] with a modified bacterial inoculum density of McFarland 0.5 and using LB broth as nutritional medium. For EOP analysis, a tenfold dilution series of the corresponding phage was prepared and tested on the original *E. coli* E28 and the re-isolate. A more than 100-fold reduction of plaques compared with the original *E. coli* E28 was classified as reduced susceptibility. For the phages AB27 and TB49, only 12 isolates from the control and 46 isolates from the experimental group were additionally tested using EOP, while for the other phages, all re-isolates that showed no plaques in the microplate test were analyzed.

For re-isolates of the four-phage trial, the overlay method was used for susceptibility testing of all phages, as described previously [55]. Then, 100 µL of a phage suspension (containing log_10_ 4 PFU/mL) and 100 µL bacterial suspension of the re-isolate (McFarland 0.5) were plated in an LB overlay agar (0.7% agar agar). If plaque formation on an isolate was reduced more than 100-fold compared with the original *E. coli* E28, isolates were classified as reduced susceptibility. Overall, 50 re-isolates from the control and 65 re-isolates from the experimental group were tested for the four-phage trial, resulting in 575 overlay tests.

#### 4.2.6. Carbon Source Utilization Testing

Re-isolates were analyzed for changes in nutrient metabolism compared with the original *E. coli* E28 isolate. The assay was performed in a 96-well GN2 MicroPlate^TM^, testing the utilization of 95 carbon sources and using an OmniLog^TM^ incubator (Biolog Inc., Hayward, CA, USA), as described elsewhere [25]. Each re-isolate was tested in duplicate. The original *E. coli* E28 was tested in four replicate tests. Sixteen *E. coli* E28 re-isolates of both trials that showed different phage-susceptibility patterns were selected for these tests. In addition, seven *E. coli* were selected, which were obtained from TBX agar plates and did not show phenotypic characteristics of *E. coli* E28 (no growth on supplemented TBX agar and no ESBL phenotype). Substrates with inconsistent utilization results for *E. coli* E28 were excluded from the data analysis. This applied for gentiobiose, D-psicose, formic acid, L-alaninamide, L-histidine, and L-ornithine. Means of the duplicate measurements with more than ±1.0 difference were considered as increased or decreased utilization compared with *E. coli* E28.

#### 4.2.7. Growth Curves

Growth of the re-isolates and E28 in LB medium was analyzed using a TECAN Infinite^®^ 200 PRO microplate reader (Tecan Trading AG, Switzerland). Every 15 minutes, the absorbance at 600 nm was automatically measured for 24 h at 37 °C.

#### 4.2.8. Data Analysis

The necessary sample size was calculated by using the Power-and-Sample-Size Tool Version 12.1 of the statistic set SAS 9.3 in cooperation with the Department of Biometry, Epidemiology, and Information Processing of the University of Veterinary Medicine Hannover, Foundation. A standard deviation of 1.8 was estimated from previous studies and a detection level of 2 log_10_ was used. Standard values were used for α and β (α = 0.05, β = 0.20). Two additional animals were housed in each group, considering the possibility of losses.

Expected phage counts in fecal samples (*E*) were calculated by dividing the estimated daily phage intake per animal by the total intake on the fattening day. Phage intake per animal was calculated as a function of daily drinking water consumption (*w*) and phage concentrations in drinking water (*p*), while the total intake of chickens was calculated by multiplying daily drinking water consumption and daily feed intake (*f*, given by the breeder [56]):

E = (p * w) / (f + w) (1)

This calculation was performed for the days on which all necessary values were measured. Drinking water consumption was calculated from the loss of water in the bell drinkers. A mean of three days was used to compensate for a varying proportion of spilled water on individual days.

Tests on normal distribution revealed some sampling times without a normal distribution of data. Thus, significances of differences between the control and experimental group were tested using a Wilcoxon rank sum test. All tests were done using SAS Enterprise guide 7.1. software.

## 5. Conclusions

The results of the two trials indicated that specific reduction of one strain in the multi-strain environment of commensal *E. coli* in the chicken gut might require a very well targeted phage selection that also considers the interaction with the commensal *E. coli*. However, the results of the trials presented here can improve the understanding of the effect of phages on single strains in the multi-strain microbiota of the chicken gut.

## Figures and Tables

**Table 1:** Mean bacterial counts in feces (log10 colony forming units (CFU)/g feces ± SEM). dph = days post hatch (Supplementary Materials).

Six-Phage Trial		6 dph	7 dph	9 dph	14 dph	21 dph	28 dph	35 dph
*E. coli*	control	8.0 ± 0.1	6.2 ± 0.5	6.2 ± 0.3	6.6 ± 0.3	6.7 ± 0.3	6.3 ± 0.3	6.5 ± 0.4
	phage group	8.5 ± 0.1	7.1 ± 0.3	7.6 ± 0.2	6.7 ± 0.5	6.8 ± 0.2	6.9 ± 0.2	6.8 ± 0.2
Model strain E28	control	8.4 ± 0.1	6.1 ± 0.3	4.2 ± 0.4	4.0 ± 0.4	3.9 ± 0.4	3.7 ± 0.3	3.7 ± 0.4
	phage group	7.7 ± 0.5	6.6 ± 0.3	6.7 ± 0.2	5.6 ± 0.3	5.2 ± 0.3	5.6 ± 0.4	5.8 ± 0.3
Four-phage trial		6 dph	8 dph	-	15 dph	22 dph	29 dph	36 dph
*E. coli*	control	6.5 ± 0.3	6.5 ± 0.4	-	6.3 ± 0.3	6.5 ± 0.3	7.0 ± 0.2	5.9 ± 0.3
	phage group	1.7 ± 0.3	3.6 ± 0.5	-	6.0 ± 0.2	5.8 ± 0.2	6.0 ± 0.2	6.0 ± 0.3
Model strain E28	control	0.0 ± 0.0	0.4 ± 0.2	-	2.7 ± 0.4	4.1 ± 0.4	5.7 ± 0.4	3.8 ± 0.4
	phage group	0.0 ± 0.0	2.6 ± 0.7	-	4.9 ± 0.3	5.2 ± 0.2	5.1 ± 0.3	5.3 ± 0.4

**Table 2:** Mean phage counts in feces compared with expected excretion without phage replication and intake dose in the phage group.

Six-Phage Trial	6 dph	7 dph	9 dph	14 dph	21 dph	28 dph	35 dph
Phage counts (log10 PFU/g feces)	7.3	6.1	4.8	n.d. ^c^	4.1	n.d. c	5.1
*Exp. without replication (max. log10 PFU/g feces) ^a^*		*4.1*		*4.7*		*4.1*	
Intake dose (log10 PFU/mL water) ^b^	n.d. c	4.2	n.d. ^c^	4.8	n.d. ^c^	4.2	n.d. ^c^
Four-phage trial	6 dph	8 dph	-	15 dph	22 dph	29 dph	36 dph
Phage counts (log10 PFU/g feces)	n.d. c	3.5	n.d. ^c^	6.4	6.9	6.3	6.3
*Exp. without replication (max. log10 PFU/g feces) ^a^*				^*d*^	*7.1*	*6.8*	
Intake dose (log10 PFU/mL water) ^b^	n.d. ^c^	n.d. ^c^	n.d. ^c^	5.6	7.3	7.0	n.d. ^c^

^a^ Estimated by the water loss of the drinkers per bird, the phage dose in the drinkers and daily intake of feed (g), given by the performance objectives for Ross 308 broilers. ^b^ The intake dose of phages per mL water was calculated as a mean of measured phages concentrations in drinking water at the indicated days. ^c^ No samples or data were obtained for the indicated days. ^d^ In the four-phage trial 15 days post hatch, water intake volume was not available and expected excretion could thus not be calculated. dph = days post hatch; PFU = plaque forming units.

**Table 3:** Proportion of E28 re-isolates showing reduced phage susceptibility (%).

Six-Phage Trial ^a^	Cocktail	EW2	KRA2	TB49	AB27	TriM	G28
Phage group	2	12	10	0	4	2	0
Control	0	0	0	2	0	0	0
Four-phage trial ^b^	Cocktail	EW2	-	TB49	AB27	-	G28
Phage group	0	20	-	10	7	-	0
Control	0	0	-	4	0	-	0

^a^ Reduced efficiency of plating (EOP, serial dilution) by a minimum of 2 log_10_ compared with the original E28. ^b^ Reduced plaque formation in overlays by a minimum of 2 log_10_ compared with the original E28.

**Table 4:** Growth, phage susceptibility pattern, and metabolic characteristics of selected re-isolates.

Characteristics				
Isolate	Phage Susceptibility Pattern ^b^	Reduced Utilization Carbon Sources (%) ^c^	Increased Utilization Carbon Sources (%) ^c^	Growth Curve in Broth (24 h, OD 600 nm) ^d^
Wildtype E28	-	-	-	>1; <1.2
Re-isolates six-phage trial				
134 ^a^	-	-	-	>1; <1.2
154	-	-	-	>1; <1.2
183	(TB49)	-	1	>1; <1.2
116	(TB49)	38	7	<1
179	EW2, (TB49), (KRA2)	-	2	>1; <1.2
185	TriM, AB27, EW2, KRA2	-	1	>1; <1.2
210	TriM, AB27, EW2, KRA2	-	2	>1; <1.2
156	TriM, AB27, EW2, TB49, KRA2	8	22	<1
Re-isolates four-phage trail				
22 ^a^	-	-	1	>1; <1.2
16	-	-	1	>1; <1.2
106	(TB49)	-	1	>1; <1.2
120	(TB49)	-	1	>1; <1.2
118	TB49	-	-	>1; <1.2
178	AB27, EW2	-	1	>1; <1.2
198	AB27, EW2	1	1	>1; <1.2
196	AB27, (EW2), (TB49)	2	1	<1
Non-E28 six-phage trial				
617 ^a^	TriM, AB27, EW2, TB49, KRA2, (G28)	-	24	>1; <1.2
630 ^a^	TriM, AB27, EW2, TB49, KRA2, (G28)	1	17	>1.2
640 ^a^	TriM, AB27, EW2, TB49, KRA2, (G28)	2	13	>1.2
650 ^a^	TriM, (AB27), EW2, TB49, KRA2, G28	1	19	>1.2
Non-E28 four-phage trial				
253	AB27, EW2, TB49, G28	-	27	>1.2
283	AB27, (EW2), TB49, G28	-	26	>1.2
311	AB27, (EW2), TB49, G28	-	26	>1; <1.2

^a^ Isolates from the control group samples. ^b^ Phage acronyms in brackets indicate a reduction of susceptibility by a minimum of two orders of magnitude compared with the original E28, acronyms without breaks indicate total resistance (no plaques). Re-isolates of the six-phage trial were tested by efficiency of plating, and re-isolates of the four-phage trial were tested with suspensions containing log_10_ 4 PFU/mL using the overlay technique, as described in Section 4. ^c^ Carbon source utilisation by the E28 re-isolates from supplemented tryptone bile X glucuronide (TBX) plates and by the non-E28 from TBX plates was tested using Biolog Phenotype Microarray GN2. ^d^ Growth curves were determined using a TECAN reader.

## Supplementary Materials

The following are available online at https://www.mdpi.com/2076-0817/9/4/293/s1, Table S1: Bacterial counts.
