# Supplementary material for: Impact of Bacteriophage-Supplemented Drinking Water on the E. coli Population in the Chicken Gut
Source: Pathogens. 2020 Apr 16;9(4):293. doi: 10.3390/pathogens9040293 (PMC7238078; doi:10.3390/pathogens9040293)
Supplement: Supplementary file 1 [file pathogens-09-00293-s001.pdf]

Table S1 Mean bacterial counts in feces (log10 CFU/g feces±SD)

**Trial 1**		**6 dph**	**7 dph**	**9 dph**	**14 dph**	**21 dph**	**28 dph**	**35 dph**
Mean *E. coli*	control	8.0±0.5	6.2±2.2	6.2±1.0	6.6±1.0	6.7±1.0	6.3±1.2	6.5±1.4
	test group	8.5±0.4	7.1±1.1	7.6±0.8	6.7±2.0	6.8±0.7	6.9±0.8	6.8±0.7
Mean E28	control	8.4±0.2	6.1±1.3	4.2±1.6	4.0±1.7	3.9±1.5	3.7±1.1	3.7±1.7
	test group	7.7±2.1	6.6±1.0	6.7±0.9	5.6±1.1	5.2±1.0	5.6±1.5	5.8±1.1
**Trial 2**		**6 dph**	**8 dph**	-	**15 dph**	**22 dph**	**29 dph**	**36 dph**
Mean *E. coli*	control	6.5±1.1	6.5±1.4	-	6.3±1.1	6.5±1.1	7.0±0.8	6.0±1.0
	test group	1.7±1.3	3.6±1.8	-	6.0±0.8	5.8±0.8	6.0±1.0	6.0±1.2
Mean E28	control	0.0±0.0	0.4±0.7	-	2.7±1.4	4.1±1.5	5.7±1.4	3.8±1.4
	test group	0.0±0.0	2.6±2.3	-	4.9±1.2	5.2±1.0	5.1±1.0	5.3±1.5
